# Effect of denture cleansers on color stability and surface roughness of denture bases fabricated from three different techniques: Conventional heat‐polymerizing, CAD/CAM additive, and CAD/CAM subtractive manufacturing

**DOI:** 10.1002/cre2.763

**Published:** 2023-07-12

**Authors:** Mehrab Takhtdar, Nahid Azizimoghadam, Mohammad Hasan Kalantari, Mina Mohaghegh

**Affiliations:** ^1^ Department of Prosthodontics, School of Dentistry Shiraz University of Medical Sciences Shiraz Iran

**Keywords:** CAD/CAM denture base materials, color stability, denture cleansers, surface roughness

## Abstract

**Purpose:**

Denture base materials are commonly exposed to different denture cleansers which can affect their essential properties. This study aimed to assess the effect of denture cleansers on color stability and surface roughness of poly methyl methacrylate (PMMA) denture bases fabricated from different techniques: Conventional heat‐polymerizing, CAD/CAM additive and CAD/CAM subtractive.

**Materials and Methods:**

In this in vitro study, 24 disc‐shaped specimens were fabricated by the mentioned methods for each group. The initial color and surface roughness of specimens were recorded. The specimens were randomly divided into three subgroups (*n* = 8): a control subgroup (distilled water), and two experimental subgroups of bioactive oxygen tablet (Corega) and 1% sodium hypochlorite solution. Then they were immersed in the solutions simulating 180 days of clinical use. Color change (∆E) was measured by a spectrophotometer according to the CIE L*a*b* color space and the American National Bureau of Standards (NBS = 0.92 × ∆E). Final surface roughness was recorded by a profilometer and its change was calculated. The Kruskal–Wallis test followed by the Wilcoxon signed rank test were used for statistical analyses (*α* = .05).

**Results:**

The conventional and CAD/CAM subtractive groups showed an increase in surface roughness following immersion in hypochlorite solution (*p* < .05). After immersion in the solutions, the highest surface roughness was noted in the conventional group, and the lowest in the CAD/CAM additive group. The CAD/CAM additive group experienced a significant color change in hypochlorite solution (*p* < .05) and showed the highest color change while the CAD/CAM subtractive group showed the lowest color change in all denture cleanser solutions.

**Conclusion:**

Although the CAD/CAM additive denture base resins had the lowest surface roughness after exposure to denture cleanser solutions, they showed significant color change, which should be taken into account. Using hypochlorite as a denture cleanser should be cautioned due to its negative effects on the surface roughness and color stability of denture base materials.

## 1 INTRODUCTION

Poly methyl methacrylate (PMMA) resin was introduced to dentistry in 1930, and it is still widely used as a denture base material due to its various advantages including superior esthetics, easy processing and repair, and cost‐effectiveness (Nandal et al., 2013).

Conventionally, dentures can be processed through compression molding, pouring a fluid resin, and injection molding (Zarb, 2013). The popularity of computer‐aided design and computer‐aided manufacturing (CAD/CAM) technology in dentistry and its potential to revolutionize the conventional approaches led to development of two novel denture fabrication techniques, namely the additive and subtractive techniques. In the subtractive milling technique, the denture base is fabricated from pre‐polymerized PMMA blocks. More recently, denture base is fabricated by 3D‐printing of the object by layer‐by‐layer addition of the desired material in additive manufacturing technique. The 3D printing method is highly cost‐effective due to its precision in creating minute details in complex structures and material waste reduction (Gu et al., 2020).

The CAD/CAM dentures have become a quickly developing part of the dental profession. They have several advantages such as simplicity, accuracy, and precision compared with the conventional denture fabrication technique. Moreover, the communication between the dentist, patient, and dental technician is improved due to the digital and easily modifiable nature of the process, with the ability to save and store the whole design as a single file for future re‐uses (Shin et al., 2020).

Despite a number of advantages, PMMA denture base materials are susceptible to bacterial colonization, which can lead to subsequent infection. Regardless of the adopted denture fabrication technique, denture plaque can cause opportunistic infections and denture‐related stomatitis (Machado et al., 2009; Nikawa et al., 1998).

Elderly and disabled patients, those in nursing homes, and even regular denture wearers may find it beneficial to use chemical cleansing methods in conjunction with mechanical cleaning. Denture cleansers have different mechanisms of action and can aid in removing the stains, debris, and biofilm from the denture surface (Paranhos et al., 2007; Saraç et al., 2007). Chemical denture cleaning methods include immersion of denture in various cleansing solutions such as sodium hypochlorite and sodium perborate. These solutions can clean the undercuts that are difficult to access mechanically (de Souza et al., 2009). However, recent studies have shown that some acrylic properties, such as color, surface roughness, and hardness, may be adversely affected by chemical solutions, regardless of the method of fabrication (Atalay, 2022; Davi et al., 2010; Porwal et al., 2017). The rough surface of PMMA offer a favorable niche to retain microbial plaque and stain. Increasing the accumulation of biofilm on the acrylic prosthesis, predominantly compounded of Candida species and the bacterial pathogens responsible for “denture stomatitis” (Atalay, 2022; Peracini et al., 2010). Stains accumulation due to eating colored foods and beverages over time cause color change in removable acrylic prosthesis. This is a cause of patient dissatisfaction due to the deterioration of aesthetic quality which makes the patient to replace the denture sooner (Saraç et al., 2007). Also rough surfaces are more susceptible to microbial colonization, plaque accumulation and discolouration. Thus surface roughness is an important physical property of acrylic resins which may be adversely affected by denture cleansers (Sahin et al., 2016; Sharma et al., 2017; Williams & Lewis, 2000).

Assessment of color stability can provide important information about the durability and maintenance of denture base materials. As mentioned earlier, exposure to disinfecting agents which can change the restoration surface, and aging due to exposure to oral fluids over time are some of the main factors causing denture base color change. Therefore, the selected cleaning technique for removable dentures should be compatible with the denture base material to preserve its properties (Atalay, 2022; Dayan et al., 2019; Porwal et al., 2017; Sahin et al., 2016).

The purpose of this study was to evaluate the effect of two different denture cleansers (bioactive oxygen tablet and 1% sodium hypochlorite) on color stability and surface roughness of different denture bases fabricated from different resins by the conventional, additive (3D‐printing), and subtractive techniques. The null hypothesis was that denture cleansers cannot affect the color stability and surface roughness of denture bases fabricated from different resins by the conventional, additive, and subtractive techniques.

## 2 MATERIALS AND METHODS

After ethical approval (IR.SUMS.DENTAL.REC.1400.019), in this in vitro study, three different denture fabrication techniques were used to fabricate disc‐shaped specimens (Table 1). The number of specimens for each group was calculated according to a previous study (Alfouzan et al., 2021a; Jain et al., 2021) and G*Power software (3.1.9.4, Heinrich Heine University Düsseldorf, Germany), assuming *α* = .05 and study power of 95%. The required sample size for assessment of surface roughness and color change was calculated to be 24 in each group, and a total of 72 disc‐shaped specimens were fabricated.

**Table 1:** Characteristics of denture base resins used in this study.

Group	Material trade name	Batch number	Manufacturer	Main composition	Fabrication technique
1	ProBase Hot Lab Kit (US‐P)	531796AN	Ivoclar Vivadent, Germany	Powder: Polymethyl methacrylate, softening agent, benzoyl peroxide, pigments Liquid: Methyl methacrylate, dimethacrylate (linking agent), catalyst	Compression molding, heat‐polymerization technique
2	Asiga DentaBASE	PN/03569	Asiga, Sydney, Australia	Photopolymerized methacrylate resin	CAD/CAM additive manufacturing, 3D printing
3	PINK CAD/CAM disc BASIC	1272/2008/EC	Polidentd.o.o., Dental Products Industry, Vočja Draga 42, 5293 Volčja Draga, Slovenija	Material on the base of polymethylmethacrylate, dimethacrylates and pigments.	CAD/CAM subtractive manufacturing, milling technique

Three fabrication techniques were used: The conventional heat‐polymerized compression molding, CAD/CAM additive manufacturing (3D printing), and CAD/CAM subtractive manufacturing (milling technique).

## 3 SPECIMEN FABRICATION

### 3.1 Heat‐polymerized conventional PMMA discs

**Figure 1:**
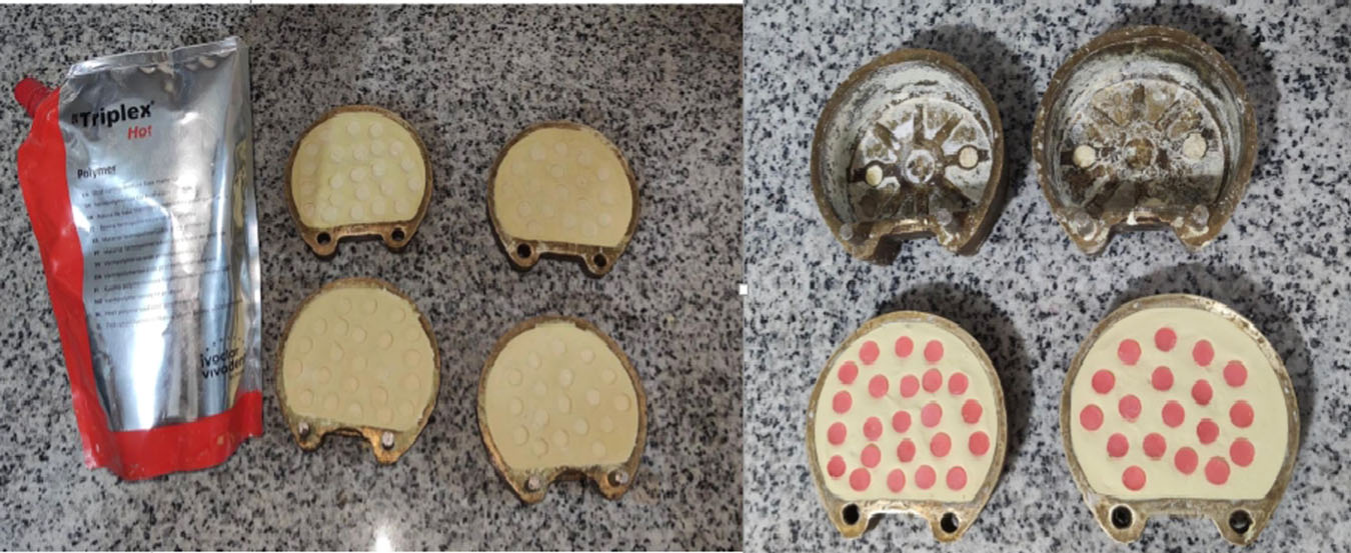
Heat‐polymerized conventional PMMA discs. PMMA, poly methyl methacrylate.

A total of 24 disc‐shaped wax patterns (10 × 2 mm) were fabricated using metal molds. The wax patterns were embedded in type IV dental stone (fujirock EP, GC) in metal flasks (61B; Two Flasks Compress, handler Manufacturing). After wax burn‐out, two layers of separating medium were applied. Mixing and packing of heat polymerizing PMMA resin (SR Triplex Hot; Ivoclar Vivadent) were performed for the conventional compression molding technique according to the manufacturer's instructions. Resins were polymerized with a long curing cycle (74°C for 8 h followed by 100°C for 1 h). Then, the flasks were allowed to cool at room temperature for 30 min. Subsequently, the joints were opened and the specimens were removed from the stone (Figure 1). The specimens were assessed for any irregularity, and defective specimens were discarded and replaced. For trimming of the specimens, a tungsten carbide bur (cross‐cut, coarse, ISO No. 500104237056, Bredent GmbH & Co., KG) was used operating at 18,000 rpm. To remove the remaining monomer, the specimens were immersed in distilled water at room temperature for 24 h.

### 3.2 CAD/CAM additive manufacturing technique (3D printing)

Each specimen was estimated to require 50 layers with 0.005 mm thickness (10 × 2 mm) and the required volume of photopolymerized methacrylate resin was 13.5764 mL (Asiga DENTABASE_007, Australia, Sydney).

**Figure 2:**
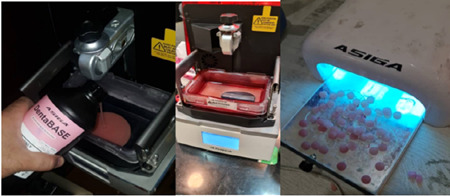
Discs fabricated by the CAD/CAM additive technique.

The STL file was transferred to CAD software (Asiga composer), and 24 specimens were printed with 0‐degree angle, without a support structure. The UV radiation intensity was 7.79 mW/cm^2^ and the irradiation time of each layer was 2.83 s. The specimen production time was calculated to be 8 min and 38 s. Then, the final file was transferred to a printer (ASIGA‐Max UV385‐Australia, Sydney) (Figure 2).

The created specimens were gently removed from the fabrication page by the part removal tool. Afterwards, they were placed in 96% isopropyl alcohol bath for 10 min. Next, they were placed at room temperature for 20 min for complete evaporation of alcohol.

Postcuring was subsequently performed with UV light (Lumamat100; Ivoclar/Vivadent) with a radiation intensity of 7.79 Mw/cm^2^, 385 nm wavelength, and 80°C temperature for 8 min and 30 s.

### 3.3 CAD/CAM subtractive manufacturing (milling) technique

Prepolymerized PMMA denture base (Polydent, Vocja Draga, Slovenia) was designed and transferred to a CAD‐CAM milling machine (M1 Wet Milling Unit; Zirkonzahn) in STL files to obtain disc‐shaped specimens (10 × 2 mm).

#### 3.3.1 Finishing and polishing of specimens

Finishing and polishing of specimens were conducted with 600‐grit carbide abrasive paper (Tufbak waterproof sanding sheets; Scour Pads Pty Ltd.) under running water for 90 s, and then with 800‐grit carbide abrasive paper (Tufbak waterproof sanding sheets; Scour Pads Pty Ltd.) for 90 s and finally with a slurry of pumice and water and a cloth wheel for polishing operating at 1500 rpm for 90 s. The specimens were then rinsed under running water for 30 s and finally dried with a cloth. To standardize the results, the polishing process was performed by one operator with gentle pressure. The polishing time of all 90 specimens was the same as measured by a stopwatch. New carbide papers was used for each specimen. A total of 24 specimens were fabricated from each resin material and divided into 1 control and 2 experimental disinfection subgroups (Table 2).

**Table 2:** Denture cleanser solutions used in this study.

Subgroup	Type of cleanser	Disinfection method	Manufacturer	Main ingredients
A	Distilled water—Control group	Immersed in distilled water at 37°C for 6 months	Nabet distilled water, Pars Morvarid company, Tehran, Iran	H_2_O
B	Sodium hypochlorite	Immersed in 1% sodium hypochlorite for 6 months	Javel water, pars chemiachlor (PCC) Persian Investment Group, Khouzestan, Iran	NaClO
C	Corega tablet denture cleanser	Immersed in Corega tablet denture cleanser for 6 months	Block Drug Company, Inc., Jersey City, New Jersey, USA	Sodium bicarbonate, citric acid, potassium caroate (potassium monopersulfate), sodium carbonate, sodium carbonate peroxide, TAED, sodium benzoate, PEG‐180, sodium lauryl sulfate, VP/VA copolymer, aroma, subtilisin, cellulose gum, CI 42090, CI 73015, CI 19140.

#### 3.3.2 Immersion protocol

The specimens fabricated from each material were randomly assigned to the following subgroups (*n* = 8).

Subgroup A: Control group (distilled water)

Subgroup B: 1% sodium hypochlorite

Subgroup C: Corega tablet denture cleanser

The specimens in each group were immersed in the respective solutions. For both the experimental groups, the immersion time was 3 min as recommended by the manufacturers. Next, the specimens were removed from the cleansing solution, rinsed with running water, and immersed again in a fresh solution for the next cycle. Thirty 3‐min immersion cycles were applied each day for 6 days to simulate 180 days of denture immersion in cleansing solutions in the clinical setting. The specimens were stored in distilled water at room temperature between the immersion cycles (Jain et al., 2021).

#### 3.3.3 Surface roughness measurement

The surface roughness (Ra) was measured by contact profilometry using a profilometer (TESA. SA, Rugosurf). The surface roughness was measured with an accuracy of 0.001 μm, a cut‐off value of 0.8 mm, and cut off length of 2 mm. The readings were performed at three points for each specimen before and after polishing. The mean of the three values was calculated and recorded as the Ra of the specimen surface. All measurements were made by a single operator, and the profilometer was calibrated before each measurement.

#### 3.3.4 Color testing

A spectrophotometer (Vita‐Easyshade V, VITA Zahnfabrik) was calibrated by using a white calibration plate provided by the manufacturer before each measurement. A positioning jig was used to standardize repeated measurements. Color change (ΔE) was calculated according to the CIE L*a*b* color space. There are three parameters that define color in this system: L*, a*, and b*, which represent lightness, redness‐greenness, and yellowness‐blueness, respectively. The ΔE of each specimen was calculated using the following formula:

ΔE=[(ΔL*)2+(Δa*)2+(Δb*)2]1/2

Where a* and b* are on the chromatic scale and show redness as +a* and greenness as −a*, while yellowness is represented by +b* and blueness by −b*. Delta L*, Δa*, and Δb* display the change in L*, a*, and b* values between the initial and secondary measurements, respectively.

The National Bureau of Standards (NBS) was used to quantify the level of ΔE (Table 3) according to the following formula:

NBS unit=ΔE×0.92

**Table 3:** Critical marks of color difference according to the National Bureau of Standards (NBS).

Critical marks of color difference	Textile terms (NBS unit)
Trace	0.0–0.5
Slight	0.5–1.5
Noticeable	1.5–3.0
Appreciable	3.0–6.0
Much	6.0–12.0
Very much	>12.0
Trace	0.0–0.5

#### 3.3.5 Statistical analysis

All statistical analyses were carried out using SPSS version 23 (IBM). The Kolmogorov–Smirnov test was used to assess the normality of data distribution. Color and roughness values were analyzed and compared among the groups by the Kruskal–Wallis test. The Wilcoxon signed rank test was applied to compare before‐ and after‐treatment values in each group. A *p* < .05 was considered statistically significant.

## 4 RESULTS

The Kolmogorov–Smirnov test showed that the data were not normally distributed. Therefore, nonparametric tests were used to analyze the data.

### 4.1 Surface roughness

**Figure 3:**
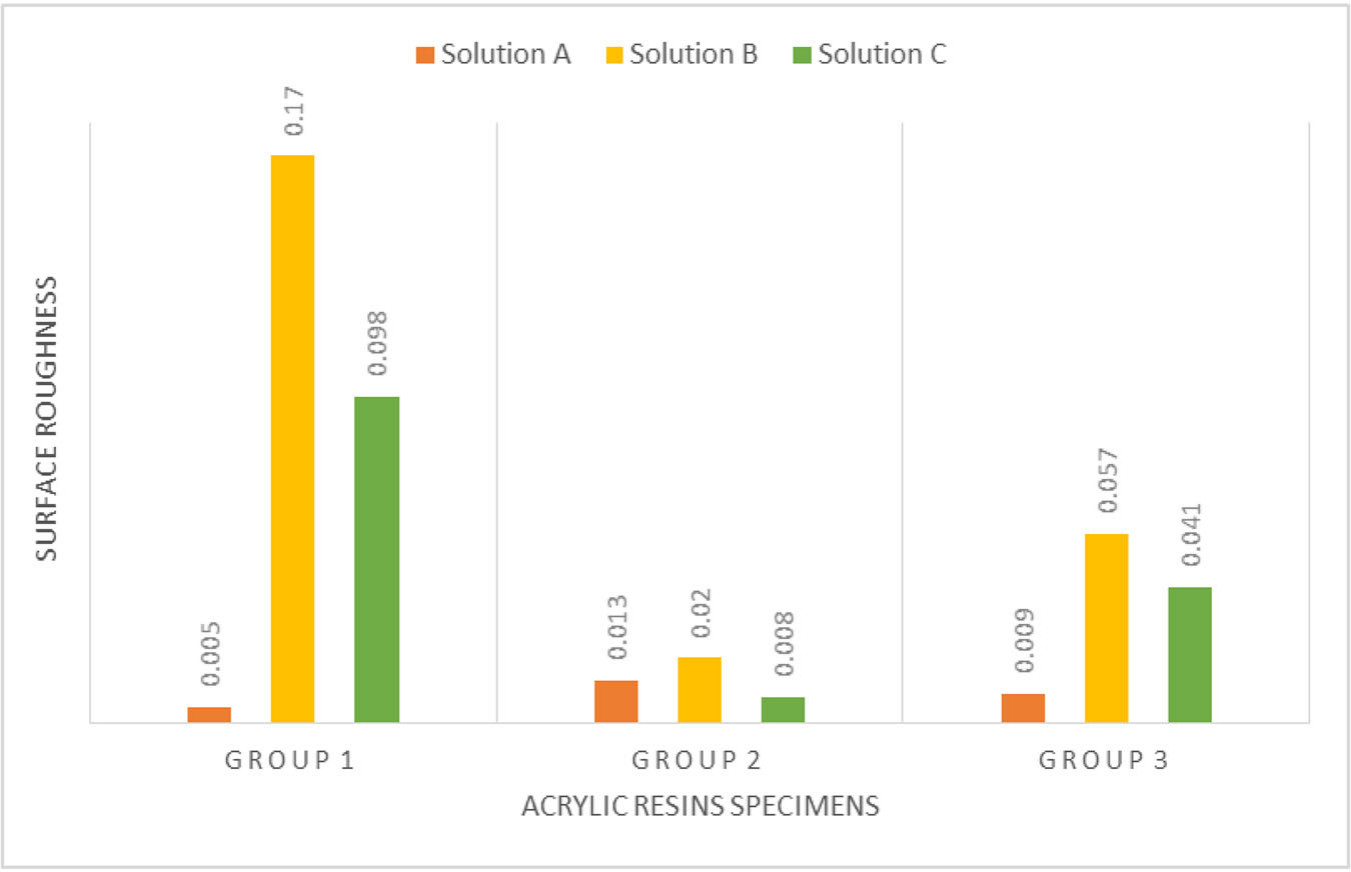
Surface roughness comparison chart of each group after testing (R2‐R1), solution A = distilled water, solution B = sodium hypochlorite, solution C = Corega tablet.

The surface roughness of specimens in the experimental groups was measured after fabrication and before immersion in the solutions. The mean surface roughness was measured at three points on each specimen surface, and the mean Ra value was calculated. As shown in Table 4 and Figure 3, group 1 (conventional PMMA discs) had the highest surface roughness.

**Table 4:** Surface roughness of the groups (*n* = 24) after fabrication (before testing).

Group	Mean	Standard deviation	Median	Minimum	Maximum
1	0.5228	0.21099	0.4885	0.16	0.94
2	0.1097	0.04352	0.1015	0.04	0.22
3	0.0732	0.03460	0.0640	0.03	0.15

*Note*: group 1 = conventional PMMA discs, group 2 = additive CAD/CAM, group 3 = subtractive CAD/CAM.

Abbreviation: PMMA, poly methyl methacrylate.

The results of Wilcoxon signed rank test regarding the surface roughness change in the three groups are shown in Table 5. A significant change in surface roughness was noted in groups 1 and 3 following immersion in hypochlorite solution (*p* < .05) (Figure 3).

**Table 5:** Comparison of surface roughness of specimens immersed in solutions A, B, and C.

Group	Solution	Delta mean (R2‐R1)	*p*‐value^a^
Group 1	Solution A	0.005	1.000
	Solution B	0.170	0.012
	Solution C	0.098	0.263
Group 2	Solution A	0.013	0.207
	Solution B	0.020	0.208
	Solution C	0.008	0.484
Group 3	Solution A	0.009	1.000
	Solution B	0.057	0.012
	Solution C	0.041	0.233

^a^ *p*‐value from Wilcoxon signed rank test.

### 4.2 Color change

The mean and standard deviation of ΔE, and color in NBS units for all groups at all time points are shown in Table 6.

**Table 6:** Mean and standard deviation of ΔE and color expressed in NBS (*n* = 8).

Groups	Subgroup	Minimum	Maximum	Median	Mean	*SD*	NBS	Intergroup comparison (∆E)^a^ *p*‐value
Group 1	Solution A	0.316	1.204	0.7545	0.710	0.316	0.65	**Group 1 vs. 2=** **S.A = 0.006** **S.B = 0.005** **S.C = 0.002** **Group 1 vs. 3=** S.A = 0.292 **S.B = 0.040** S.C = 0.430 **Group 2 vs. 3=** **S.A = 0.003** **S.B = 0.001** **S.C = 0.006**
	Solution B	0.224	1.105	0.8790	0.776	0.350	0.71	
	Solution C	0.141	1.507	0.5140	0.619	0.430	0.57	
Group 2	Solution A	0.458	2.193	1.7515	1.628	0.532	1.50	
	Solution B	0.735	2.119	1.8195	1.697	0.448	1.56	
	Solution C	1.010	1.934	1.5820	1.557	0.313	1.43	
Group 3	Solution A	0.224	1.100	0.4875	0.552	0.289	0.51	
	Solution B	0.224	0.616	0.3305	0.359	0.139	0.33	
	Solution C	0.141	1.803	0.3465	0.545	0.550	0.50	

*Note*: The bolded items in intergroup compressions refer to the items that have significant differences.

Abbreviation: NBS, National Bureau of Standards.

^a^ *p*‐value, S.A = Solution A.

**Figure 4:**
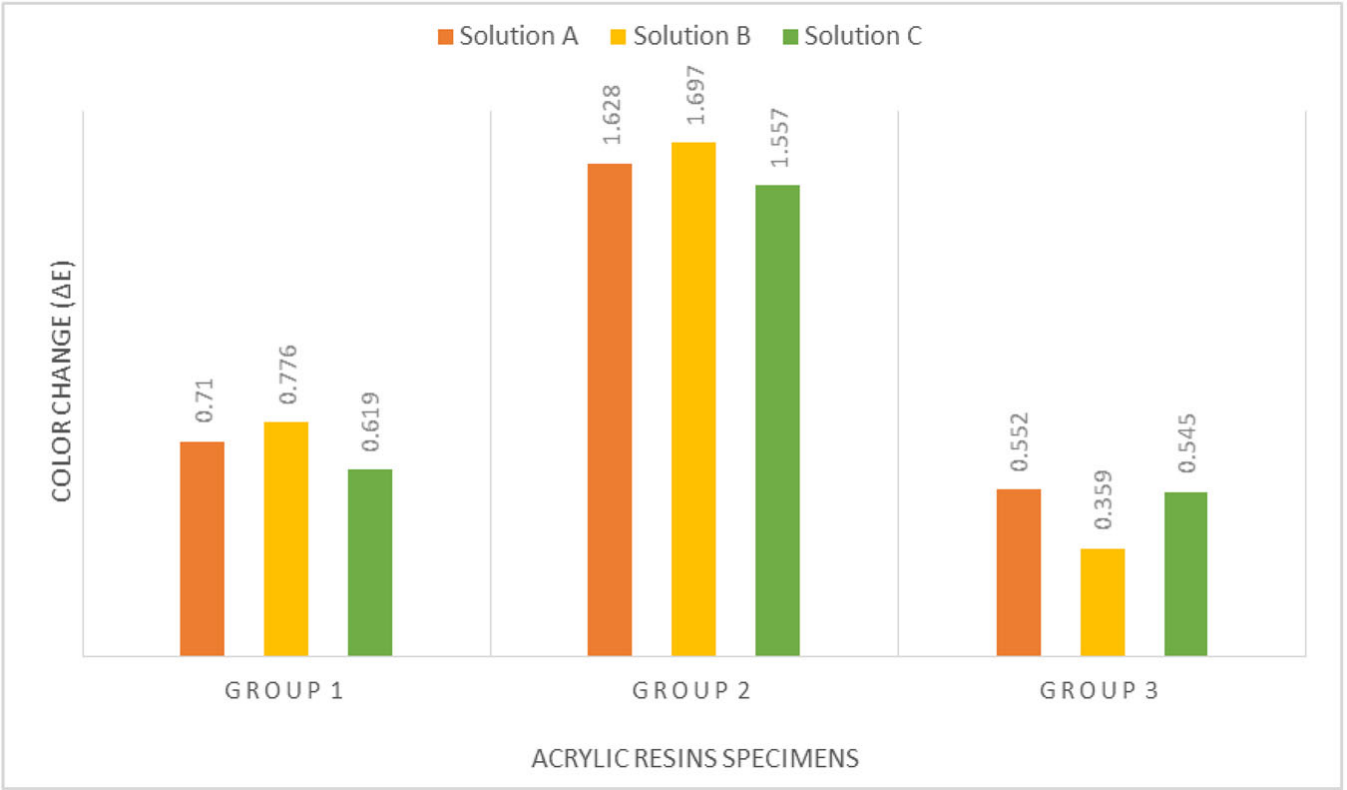
Mean ΔE comparison of groups after exposure to solutions.

The mean ΔE comparison chart of the groups is shown in Figure 4. The Kruskal–Wallis test showed that different solutions did not cause a significant color change in the specimens in the same group.

Among all solutions, group 2 caused the greatest color change (1.628 ± 0.532, 1.697 ± 0.448, and 1.557 ± 0.313, respectively) while group 3 caused the lowest color change (0.545 ± 0.550, 0.359 ± 0.139, and 0.545 ± 0.550). The magnitude of color change in group 1 was between groups 2 and 3 (0.710 ± 0.316, 0.776 ± 0.350, and 0.619 ± 0.430) (Table 6).

Despite the significant difference in ΔE between group B and both groups A and C in all solutions, only hypochlorite was able to create a significant difference between groups A and C.

In use of NBS units to evaluate color change (Table 2), the subtractive CAD/CAM acrylic resin base showed “trace” in solution B and “slight” in solutions A and C. The changes in conventional resins in all three solutions were in the “slight” range. However, changes in additive CAD/CAM acrylic resin base (group 2) in all solutions were “noticeable.”

## 5 DISCUSSION

This in vitro study evaluated the effect of two different denture cleansing materials and distilled water as the control group on the surface roughness and color stability of PMMA acrylic denture bases fabricated by three different methods after an immersion period simulating 6 months of clinical usage. The null hypothesis that the denture cleansers cannot affect the surface roughness and color stability of denture base materials was rejected.

Surface roughness is a critical property of resin materials, which directly enhances the risk of oral bacterial colonization. Generally, increased surface roughness decreases the cleaning efficacy of denture base and increases bacterial accumulation on denture surface (Rapone et al., 2022).

Two different types of profilometry have been documented: contact profilometry where a metallic probe scans the surface, and laser or optical or noncontact profilometry, where a source of light is used to scan the sample surface and the light beam diffracted by the surface roughness is collected on a mirror (Mabilleau & Sabokbar, 2008). In the present study, we have used a contact profilometer to measure the surface roughness.

As shown in Table 6, after fabrication of specimens, the conventional PMMA (group 1) showed the highest surface roughness followed by additive CAD/CAM resins (group 2), and the minimum surface roughness was noted in the subtractive CAD/CAM resins (group 3). Lower surface roughness in group 3 after fabrication may be attributed to the polymerization of blocks under high pressure and temperature. However, layer by layer addition of resin in group 2, and manual mixing of resin in group 1 may create voids in resin discs and increase their surface roughness.

After immersing the specimens in the solutions for 180 days, the lowest level of surface roughness was noted in group 2 and as seen in Figure 4, all solutions increased the surface roughness in all three groups. The difference in surface roughness compared with the initial mean value was not significant among the groups.

In comparison of solutions, only sodium hypochlorite caused a significant change in surface roughness of both the conventional and subtractive CAD/CAM groups. A systematic review has discussed the effect of chemical cleansers, including sodium hypochlorite, on surface roughness of denture base materials (Schwindling et al., 2014). In this systematic review, 14 articles reported a change in surface roughness after immersion in sodium hypochlorite at concentrations ranging from 0.05% to 5.15%. Two of these 14 articles reported a decrease in surface roughness (De Rezende Pinto et al., 2010; Lira et al., 2012) and three of them, similar to the present result, reported an increase in surface roughness (Carvalho et al., 2012; Odagiri et al., 2012; Paranhos et al., 2013).

Consistent with the present study, Porwal et al., 2017, immersed several denture base resin materials (conventional thermal resin, high impact resin and polyamide denture base resin) in two denture cleansers (sodium perborate and sodium hypochlorite) for 180 days. Measuring of surface roughness with contacting profilometer before and after test showed all specimens had a significant change in surface roughness, and the highest change was noted in the conventional heat‐cure resin immersed in sodium hypochlorite.

However, the present results were different from the findings of Gad et al., 2022, who assessed the flexural strength, impact strength, hardness, and surface roughness of 3D‐printed denture base resin in comparison with heat‐cured resins. The surface roughness in their study measured by noncontact profilometer. The specimens were radially scanned 5 times at different points with 0.01 mm resolution and the average surface roughness for each specimen was calculated. They showed significant differences in all evaluated properties between the heat‐polymerized and 3D‐printed denture base materials. The 3D printed resin had inferior flexural strength, impact strength, and hardness values than the heat‐polymerized resin, while it demonstrated superior surface roughness. However, the mean Ra values for each group after testing, were smaller than the plaque accumulation threshold of 0.2 µm and lower than the clinically unacceptable threshold of 10 µm (Taşın et al., 2022).

A finite size of probe tip distorts a surface profile to some degree. It can be caused by probe load. A sharp probe head even under low loads may be have a local pressure sufficiently high to cause significant local elastic or plastic deformation of the surface being measured or make a visible scratch on softer surfaces. Therefore, the normal loads have to be low enough so that the contact stresses do not exceed the hardness of the surface to be measured. Otherwise, the contact probe can increase the surface roughness and overestimate the results. Another error may be occurred by the kinematics of the probe movement on the surface and the possibility of separation from the surface and lack of contact in some areas. It is clear that a trace for which probe contact has not been maintained presents inaccurate information about the surface micro‐roughness (Bhushan, 2000). Optical or noncontact profilometry is a more recent and modern approach and has been developed to increase accuracy. With this technique, it is possible theoretically to assess the roughness as low as a nanometer. However, the limitation of this technique is to have a surface capable of reflecting the light beam and often it is necessary to modify the sample surface to obtain a better reflection (Mabilleau & Sabokbar, 2008).

Accordingly, it is suggested that in future studies, to measure the surface roughness, should select the appropriate profilometer for the surface.

This study also evaluated the effects of denture cleansers on color stability of the three groups of denture base resins. A significant color change was noticed after comparison of the three groups. However, the magnitude of color difference varied based on the fabrication technique and type of denture base material.

The color of denture resins in this study was evaluated based on the CIE L*a*b* color space using a UV light visible spectrophotometer. Spectrophotometry is the most commonly used accurate method for this purpose since it enables quantitative measurement of a material's color change in addition to objective assessment of color (Alfouzan et al., 2021a).

Perceptual color change in dental materials can be assessed according to the CIE L*a*b* color space with a scale that contains all colors visible to the human eye. The instrumental color value readings have advantages over subjective visual color readings since they are objective, quantitative, and more easily available. In dentistry, the CIE L*a*b* 50:50% perceptibility threshold and 50:50% acceptability threshold are considered to be ΔE = 1.2 and ΔE = 2.7, respectively (Alfouzan et al., 2021b). In the present study, the ΔE values were far below the clinical acceptability threshold for each of the three denture base resins after immersion in denture cleansers.

For the acrylic resins evaluated in the present study, the values of L*, a*, and b* decreased in all solutions. Hong et al. (2009) suggested that this finding may be due to water sorption and solubility of the materials. They investigated the effect of denture cleansers on color stability of three different types of acrylic resins (heat‐polymerized, auto‐polymerized, and visible light‐polymerized) and similar to the present results, they concluded that the ∆E values of all denture base acrylic resins increased with time (Hong et al., 2009). This study also revealed that in all three cleansing solutions, additive CAD/CAM denture base resin showed the highest color change, and subtractive CAD/CAM denture base resin showed the lowest color change, while the color change of the conventional group was between these two groups. This finding was in agreement with the results of Alfouzan et al. (2021a, 2021b). In their in vitro study, the color stability of heat‐cured, light‐cured, and prepolymerized CAD/CAM acrylic resin base materials was measured after exposure to mechanical brushing and immersion in chemical denture cleansers (Corega, 5.25% sodium hypochlorite, and 0.2% chlorhexidine gluconate). The results showed that the color stability of the pre‐polymerized CAD/CAM acrylic discs was comparatively superior to the conventional acrylic resin materials. Multiple factors including water sorption, stain accumulation, degradation of intrinsic pigments, dissolution of ingredients, foods, beverages, chemical disinfectants, and surface roughness can affect the color stability of denture base materials. Besides, color is affected by the resin matrix composition and its polymerization process (Alfouzan et al., 2021b).

The current results were also in agreement with the findings of previous studies. Gruber et al. (2021) evaluated the color stability of three types of PMMA resins (CAD/CAM subtractive, additively manufactured (3D‐printed), and conventional heat‐cured resins). The results showed maximum discolouration and inferior color stability of 3D‐printed resin groups in comparison with the CAD/CAM subtractively manufactured and conventional PMMA resins. Similarly, Shin et al. (2020) studied the color stability of CAD/CAM blocks and 3D printed resins with respect to the material type, colourant type, and duration of immersion in the colourants. The authors found that 3D‐printed resins showed a color change above the clinically accepted threshold (2.25) following storage for 7 days or longer in all experimental groups. They suggested that discolouration must be expected when using 3D printing resins for restorations.

However, the present results were different from the findings of Jain et al. (2021), who investigated the effect of two commercially available denture cleansers on color stability of denture base resins fabricated by different techniques. They reported that the highest color change occurred in the subtractive group, and the lowest color change was seen in the additive group.

There are multiple reasons for the low color stability of 3D printed resin specimens. Since 3D printing is based on the additive manufacturing technique, there are layers on the surface microstructure (Shin et al., 2020). In addition, the low polymerization rate of 3D printing resins compared with other materials is another reason for the low color stability. Conventional PMMA and milled resins are made by polymerization at a high‐temperature and high‐pressure environment. Therefore, the rate of polymerization in these materials is high and their structure is compact. In contrast, although 3D printing resins undergo post‐curing processes after printing, their polymerization rates are relatively low (Tahayeri et al., 2018).

As shown in Table 6 and Figure 4, the surface roughness of the unpolished surface was higher in the printed specimens than milled specimens. But after immersion in the solutions, it showed less surface roughness. Therefore, it would be difficult to explain the low color stability of 3D printed specimens based on their surface roughness.

The present study had a number of limitations. It did not perfectly simulate the clinical setting. In the clinical oral environment, dental materials are subjected to occlusal forces, pH changes, and saliva containing various proteins and enzymes. Thus, the present results should be verified in the clinical setting. Some other factors such as thermocycling, exposure to ultraviolet light, brushing, and diet could also affect the color staining and surface properties of denture base. Therefore, future investigations with more precise simulation of the oral environment should be designed to evaluate the effect of these factors.

Also, this study was conducted within a 6‐month period; while, denture cleansers may be used for a much longer time. Moreover, a single type of material and shade were used in each group. Different materials and shades might yield different results. Another limitation was the number of cleansing agents. The surface of specimens used in this study was flat and did not simulate the surface topography of actual dentures. In addition, the effects of other aging processes such as dynamic loading were not taken into consideration.

## CONCLUSION

Within the limitations of this in vitro study, the following conclusions can be drawn:

1‐ Despite absence of a significant difference among the groups, the highest change in surface roughness, both before and after the intervention, was noted in the conventional group.
2‐ After specimen fabrication, the surface roughness of the CAD/CAM additive group was higher than that of the CAD/CAM subtractive group, but after immersion in the solutions, the CAD/CAM subtractive group showed a greater change in surface roughness.
3‐ Sodium hypochlorite significantly increased the surface roughness in the conventional and subtractive CAD/CAM groups.
4‐ In all solutions, the color change of the additive CAD/CAM group was significantly higher than the conventional and subtractive groups. This color change was “noticeable” in the NBS unit that should be taken into account when using these materials as denture base.

## AUTHOR CONTRIBUTIONS

**All co‐authors**: contributed to study design, data collection and study execution, data analysis and interpretation, and preparation of the manuscript.

## CONFLICT OF INTEREST STATEMENT

The authors declare no conflicts of interest.

## Data Availability

Data for this study are available from the corresponding author upon request.
